# Performance of eight serum cytokine/chemokine biomarkers in discriminating between active and latent tuberculosis infection in Ghana

**DOI:** 10.3389/fimmu.2025.1600712

**Published:** 2025-06-06

**Authors:** Gloria Ivy Mensah, Jones Amo Amponsah, Nana Boakye Alahaman, Isaac Anim-Baidoo, John K. A. Tetteh, Kennedy Kwasi Addo, Kwadwo Ansah Koram

**Affiliations:** ^1^ Department of Bacteriology, Noguchi Memorial Institute for Medical Research, University of Ghana, Accra, Ghana; ^2^ Department of Immunology, Noguchi Memorial Institute for Medical Research, University of Ghana, Accra, Ghana; ^3^ Department of Clinical Microbiology, School of Medicine, University for Development Studies, Tamale, Ghana; ^4^ Department of Medical Laboratory Sciences, School of Biomedical and Allied Health Sciences, University of Ghana, Accra, Ghana; ^5^ Department of Epidemiology, Noguchi Memorial Institute for Medical Research, University of Ghana, Accra, Ghana

**Keywords:** cytokine, serum, active TB, latent TB, multiplex ELISA, Ghana

## Abstract

**Introduction:**

The existing Interferon γ release assay (IGRA) tests for TB infection, lacks utility in discriminating between active TB (ATB) and latent TB infection (LTBI). This study evaluated the potential of eight serum cytokines/chemokines in differentiating LTBI from ATB and as a surrogate marker for TB treatment response.

**Methods:**

We quantified and compared the serum levels of pro-inflammatory cytokines (TNF-α, IFN-γ, IL-12p70, IL-17A, Granzyme B) and anti-inflammatory cytokines (IL-10, IL-6, IL-4) among LTBI, ATB, and healthy controls using the Human Magnetic Luminex™ 200 system. Serum cytokine/chemokine levels were also assessed at four timepoints before and during TB treatment.

**Results:**

Among ATB cases, there were twice as many males (69%) as females (30%), with infectivity spanning a wide age range. IFN-γ, IL-6, IL-10, IL-4, and IL-17A levels were higher in LTBI compared to ATB. IL-12p70 was found to be a good discriminant between ATB and LTBI (21-fold increase in ATB compared to LTBI, p < 0.05) but it did not have a good predictive potential for treatment (follow up). The predictive potential of TNF-α, IL-6, IL-10, IFN-γ, IL-4, IL-17A, Granzyme B and IL-12p70 to differentiate between ATB and LTBI using AUROC was 57%, 98 %, 91%, 100%, 100%, 97%, 66% and 100% respectively.

**Discussion:**

These findings confirm reports from other studies in different settings that LTBI and ATB express differential cytokine profiles that can be exploited as diagnostic biomarkers. Of note, the quantitative estimation of IL-12p70 may serve as a valuable marker for monitoring disease progression and treatment success in tuberculosis.

## 1 Introduction

Tuberculosis is a chronic infectious disease usually caused by the bacteria *Mycobacterium tuberculosis* (MTB), one of the seven closely related members of the *Mycobacterium tuberculosis* complex. The bacteria usually persist in its host in an asymptomatic state of latency aptly described as latent tuberculosis infection (LTBI). Favored by immunosuppressive factors such as age, drugs as well as diseases from infectious and non-infectious agents, LTBI may progress to active tuberculosis (ATB) over a month to several decades (1).

Current efforts to control tuberculosis include enhanced diagnostics, access to screening tools, and successful patient care (2). Tuberculosis transmission mathematical models show that the effectiveness of active case finding as a strategy for control of TB depends on the ability to detect TB at the early or subclinical phase of infection (3). Furthermore, it is recognized that early diagnosis is the most successful method for the management of ATB, because early TB therapy accelerates bacilli clearance in infected individuals to non-infectious, therefore blocking the chain of tuberculosis transmission (4). Diagnosing and treating latent tuberculosis (TB) infection (LTBI) is an important strategy to accelerate the decline in global TB burden and achieve TB elimination by 2030 (5). Treatment with INH is limited by the perception that the risk of toxicity for treating latent tuberculosis is greater than the reactivation to ATB (6).

With no Gold standard test for LTBI, the diagnostic methods for LTBI are old tuberculin skin test (TST) (7), and the relatively new interferon-γ release assays (IGRA), commercially available as QuantiFERON-TB and the T-SPOT.TB assay. The IGRA assay has a higher sensitivity compared to the TST, especially in Bacillus Calmette–Guerin (BCG) vaccinated individuals (8, 9). These assays detect prior immune response to M. tb antigens and do not directly detect the presence of viable bacilli (10). QuantiFERON Gold test (QFT) based on the whole blood aided enzyme-linked immunosorbent assay (ELISA) and the T-SPOT.TB test based on the peripheral blood mononuclear cell (PBMC) aided enzyme-linked immunospot assay are the two IGRAs identified commercially. Both IGRAs use the 6 kDa early secretory antigen target (ESAT-6) and 10 kDa culture filtrate protein (CFP-10) encoded region of difference 1 (RD1) as antigens (11).

Mycobacterial culture or nucleic acid amplification (NAA), including Xpert MTB/RIF and loop-mediated isothermal amplification (LAMP) assays are currently the gold standard tests for diagnosing active tuberculosis (12–14). Acid-fast bacillus smear -negative patients who cannot produce sputum spontaneously are usually diagnosed by sputum induction and/or bronchoscopy (15). However, as a limitation, IGRAs do not distinguish between ATB and LTBI especially in children (16, 17). Furthermore, there is decreased sensitivity and specificity for IGRAs, showing that a single biomarker IFN-γ may not be likely to fulfill the urgent criteria for accurate distinction between ATB and LTBI (18). Therefore, in addition to IFN-γ, it is imperative to identify other cytokines/chemokines that can discriminate between ATB and LTBI as well as expand existing immune-based diagnostic tests for ATB (19).

In 2022, the WHO Africa region reported the second largest number of new cases of TB (23%) after South-East Asia (46%) (20). In addition, TB prevalence in Ghana currently stands at 286 per 100,000 population (21), a threefold increase from the previous estimate of 90 per 100,000 population. However, case detection in Ghana remains low and active case finding would be much easier with a screening assay that can distinguish between active and latent TB. Previous studies among 100 household contacts of TB cases in Accra reported a prevalence of 65% using the QuantiFERON tuberculosis gold-in-tube (QFT-GIT) test indicating a high TB infection prevalence (22).

This study aimed to evaluate the diagnostic potential of eight serum cytokines/cytokines in differentiating LTBI from ATB cases in Ghana.

## 2 Materials and methods

### 2.1 Study participants and sampling

**Figure 1:**
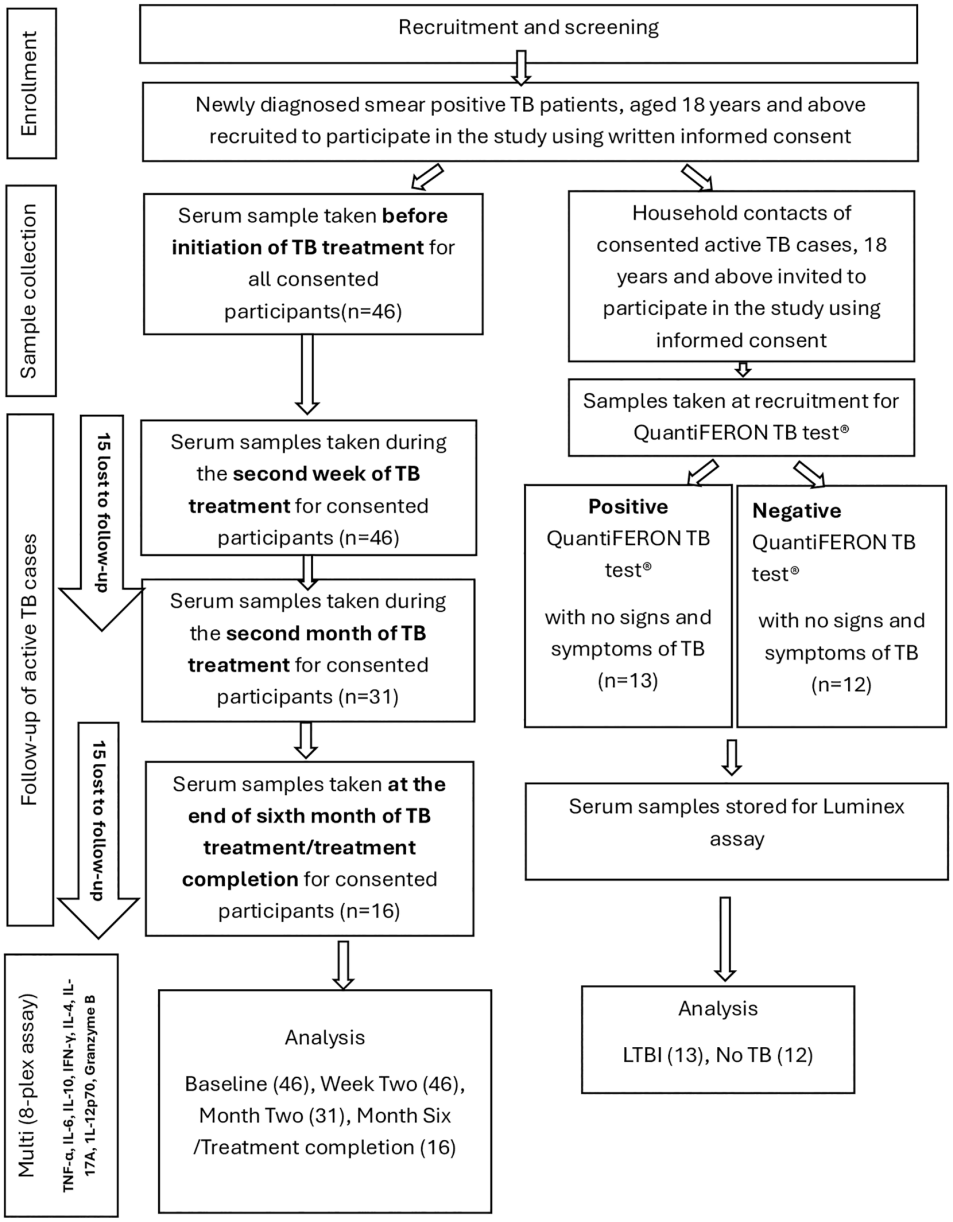
Flow chart of participant recruitment and sampling.

Serum samples from 71 participants comprising 46 active TB cases, 13 latent TB cases and 12 healthy controls with no TB infection were used. The latent and active TB cases were recruited from two primary health care facilities in Ghana: The Achimota and Maamobi General Hospital by convenient sampling. Newly diagnosed TB patients at the two facilities were introduced to the study before initiation of TB treatment. The study was explained to them in the local language or in English using the information sheet from the consent form. Those who consented to participate in the study were then asked to fill the consent form and append their signature or thumb print. The form was also witnessed by a relative of the participant. The first sample was taken prior to initiation of anti TB therapy. Household contacts of active TB cases were invited and informed about the study and those who met the edibility criteria and agreed to participate were also recruited into the study using written informed consent. All active TB cases were confirmed by a positive sputum smear microscopy using the auramine stained fluorescence microscopy technique in addition to clinical manifestations. Latent TB cases were defined as asymptomatic household contacts of recruited active TB patients with QuantiFERON-TB positive results. QuantiFERON-TB negative participants were considered as healthy controls (no TB infection) using the QuantiFERON-TB Gold test (Cellestis) and the QFT-GIT Analysis Software according to manufacturer’s instructions. In summary blood samples (2–5 ml) were collected in plain tubes. After 30–60 minutes at room temperature, samples were centrifuged at 1,500 rpm for 15 minutes and the resulting sera was stored at -80°C until analysis. Blood samples of ATB patients were taken at four (4) different time points. Serum samples were taken at baseline (before initiation of anti-TB therapy), at two weeks after initiation of therapy, at the end of the second month (intensive phase) of treatment and after 6 months or upon completion of anti-TB therapy. Of 46 active TB cases sampled at baseline, all 46 were available for sampling at (two) 2 weeks after TB treatment. However, the number reduced to 31 at two months after TB treatment and 30 were lost to follow-up at TB treatment completion. Samples from LTBI and healthy controls were taken once. ATB and LTBI participants were enrolled from a TB case-contact study (Study Number: 114/15-16). Participants less than 18 years old, relapse and ATB patients undergoing retreatment, HIV/AIDS, cancer and patients on steroid therapy were excluded from the study. Samples were blinded for all patient information except study number and sampling day until after testing. The recruitment and sampling process and timelines are shown in the flow chart (**Figure 1**).

### 2.2 Quantification of serum cytokines

Cytokine quantification was performed by multiplexing using the Luminex™ 200 system (Luminex, Austin, TX, USA) following manufacturer’s instructions.

In summary, all reagents were brought to room temperature. All standards and samples were assayed in duplicate. Microparticles (microspheres or beads) and Streptavidin-Phycoerythrin (streptavidin-PE) were always protected from light. All reagents, standards, and samples were prepared as directed by the instruction manual prior to use. A 96-well plate template was prepared for the blank, standards, and the unknown samples. Fifty microliters (50µL) of blank, standards or samples were added per well according to the prepared plate template (attachment) after which 50µL of the diluted microparticle cocktail was added to each well of the plate after vortexing. The 96-well plate was securely covered with a foil plate sealer. The plate was incubated for 2 hours at room temperature on a horizontal orbital microplate shaker set at 800 revolution per minute (rpm). With reference to the magnetic device user manual for proper washing technique, the 96-well plate was washed three times using a magnetic device designed to accommodate a microplate after 2 hours incubation. After washing, 50µL of diluted biotin-detection antibody cocktail was added to each well of the plate. The plate was securely covered with a foil plate sealer and was incubated for one hour at room temperature on an orbital plate shaker. The plate was washed three times after one hour incubation and then 50µL of diluted streptavidin-PE was added to each well of the plate. The plate was securely covered with foil plate sealer and incubated for 30 minutes at room temperature on an orbital plate shaker. After 30 minutes of incubation, the plate was washed 3 times. The microparticles were resuspended by adding 100µL of wash buffer to each well of the microplate well and the microplate was incubated for 2 minutes on an orbital microplate shaker set at 800 rpm. The plate was read within 90 minutes using a Luminex 200 analyzer.

### 2.3 Statistical analysis

Test outcomes were saved in Microsoft Excel 2020 (Microsoft Corp., Washington, USA) prior to analysis using Prism version 8.4.3 (GraphPad Software, Inc.). To compare the median cytokine/chemokine levels as well as longitudinal changes in median cytokine levels, Kruskal–Wallis or One-way ANOVA, was used for data involving three groups whereas Mann–Whitney U test was used for data comparison between two groups. Both Kruskal–Wallis and Mann–Whitney U tests were used to analyze data that were not normally distributed. To determine the cytokines that can discriminate active TB cases from latent TB infection, a Receiver Operating Characteristic (ROC) curve was derived using % sensitivity and % specificity for each cytokine and the Area Under the curve was calculated (23). Statistical significance was set at P-values < 0.05.

### 2.4 Ethical considerations

This study was approved by the Institutional Review Board of the Noguchi Memorial Institute for Medical Research (NMIMR-IRB (number: Study Number: 114/15-16), and the Institutional Review Board of the School of Biomedical and Allied Health Sciences, University of Ghana (SBAHS/AA/MLAB/10325639/2019-2020). The archived samples used for this study were from consented participants for whom the study was fully explained in a local dialect and English, and written informed consent was obtained with the information sheet clearly stating that samples would be archived for future studies. Anonymized data were used for analysis.

## 3 Results

### 3.1 Characteristics of participants

Serum samples from 71 participants were available for this study, these comprised 46 active TB cases, 13 latent TB cases (QuantiFERON TB test-positive) and 12 healthy controls with no TB infection (QuantiFERON TB test-negative). The 46 active TB cases included 32 (69.57%) males and 14 (30.4%) females with an average age of 37.7 ± 12.11 years for males and 34.9 ± 12.34 years for females. The age range was (18 – 62) years for males and (18 – 55) years for females. The 12 healthy control cases included 5 (41.67%) males and 7 (58.33%) females with an average age of 34.75 ± 28.95 years for males and 21.29 ± 8.69 years for females. The age range was (18 – 78) years for males and 18–41 years for females. The 13 Latent TB cases comprised of 3 (23.08%) males and 10 (76.92%) females with an average age of 30 ± 10.58 years for males and 29.33 ± 13.96 years for females. The age range was from 18–38 years for males and 18–58 years for females. **Table 1** shows the age and sex distributions of the study groups.

**Table 1:** Age and sex characteristics of study participants.

Groups	n (%)	Age range	Mean Age
Active TB		(Years)	
Male	32 (69.57)	18-62	37.7 ± 12.11
Female	14 (30.43)	18-55	34.9 ± 12.34
Latent TB			
Male	3 (23.08)	18-38	30 ± 10.58
Female	10 (76.92)	18-58	29.33 ± 13.96
No TB			
Male	5 (41.67)	18 – 78	34.75 ± 28.95
Female	7 (58.33)	18 – 41	21.29 ± 8.69

### 3.2 Median cytokine levels among study cohorts

**Figure 2:**
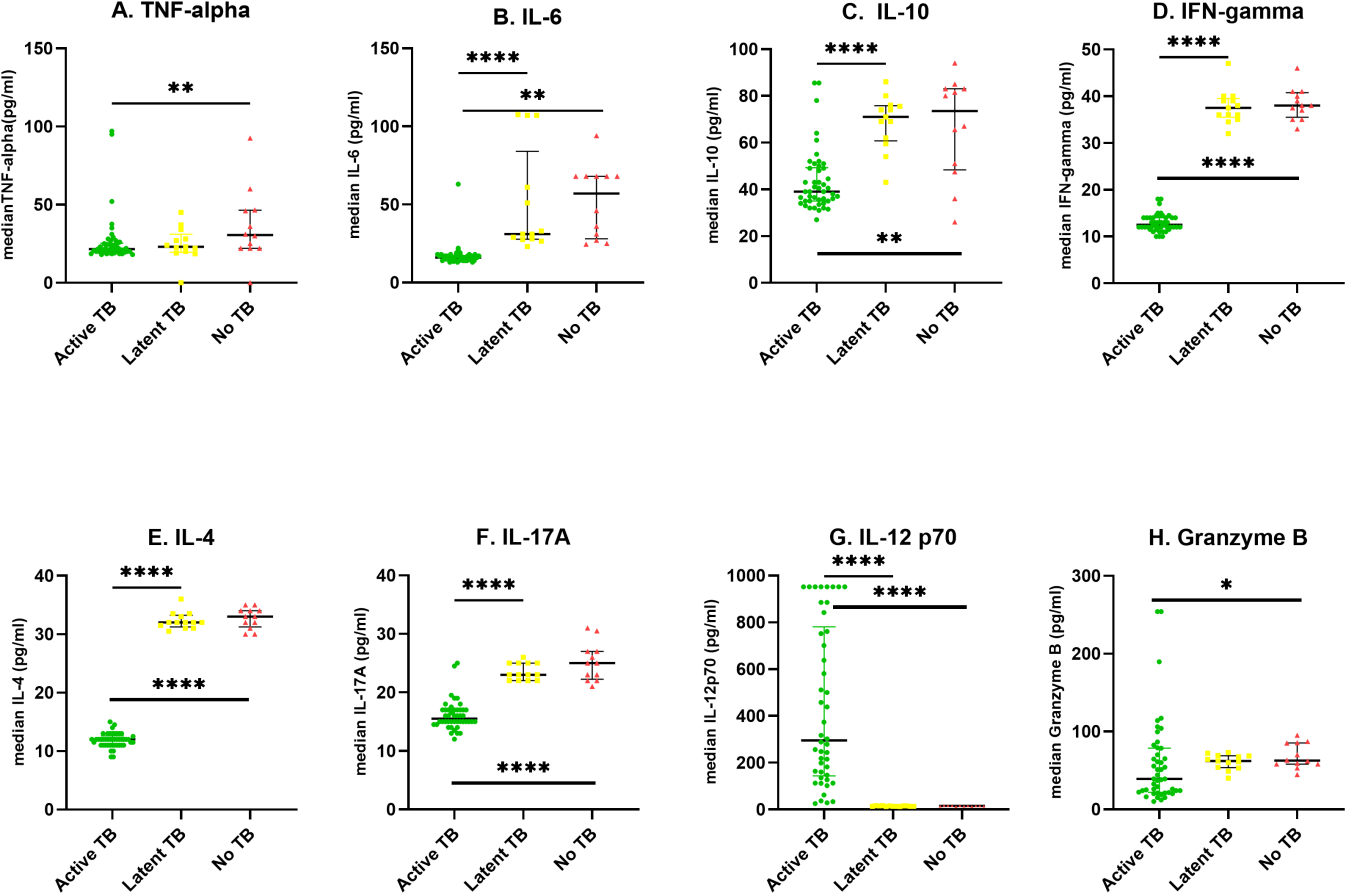
Scatter plots showing median cytokine levels (pg/ml) of the three groups; The levels of cytokines were determined in the serum of individuals with *Active TB* (ATB) n=46, *Latent tuberculosis infection* (LTBI) n=13 and *healthy controls* (No TB) n=12 in an 8-plex Luminex assay. Scatter plots represent median serum concentrations of cytokines **(A–H)** in participants with ATB (green circle), LTBI (yellow square), and No-TB (red triangle). Statistical differences between two groups were analyzed using Mann−Whitney U test *P < 0.05, **P < 0.01, and ****P < 0.0001.

The levels of each cytokine/chemokine as measured by the multiplex assay were expressed as median concentration (pg/ml). A dot plot of each cytokine/chemokine was was generated for the three independent study cohorts using Graph Pad Prism (v 8.4.3) as shown in **Figure 2**. The levels of IFN-γ, IL-6, IL-10, IL-4, IL-17A were significantly higher in LTBI patients compared to ATB cases (p ≤ 0.0001). Specifically, IFN-γ, IL-6, IL-10, IL-4, IL-17A were respectively 3, 2, 1.8, 3 and 2.5-fold higher in LTB. Only the levels of IL-12p70 were significantly lower (p ≤ 0.000) in LTBI than ATB. Of note, a 21-fold increase in IL-12p70 levels was observed in ATB compared to LTBI participants.

The ATB patients had significantly lower levels of IL-12p70 (p ≤ 0.0001), TNF-α (p ≤ 0.01), IL-6 (p ≤ 0.01), IL-10 (p ≤ 0.01), IL-4 (p ≤ 0.0001) and IL-17A (p ≤ 0.0001) compared to healthy controls representing a 20, 1.8, 3.5, 1.8, 2.8 and 1.5-fold decrease respectively. There was no significant difference in cytokine levels between LTBI, and healthy controls as shown in **Figure 2**.

### 3.3 Longitudinal changes in median cytokine levels during TB treatment

IL-4 levels decreased significantly (p=0.0079) from baseline to week two and remained low up to month two before increasing significantly at month 6. There was a steady increase in median IL-17 levels from baseline to week two (p= 0.8486), week two to month two (p= 0.4478), month 2 to month 6 (p= 0.0024), however only the increase between baseline and month 6 and that from week two to month 6 were significant (p= 0.0073 and p= 0.0301) respectively. Similarly, IL-12p70 levels increased steadily during treatment, however only the increase from baseline to month 6 (p <0.0001), week two to month 6 (p= 0.0020) and month 2 to month 6 (p= 0.0425) were significantly different. Granzyme B levels increased 4-fold from baseline (p <0.0001) and 3.8-fold from week two (p= 0.0019) and 3.3-fold from month 2 (p=0.0052) to end of treatment.

**Figure 3:**
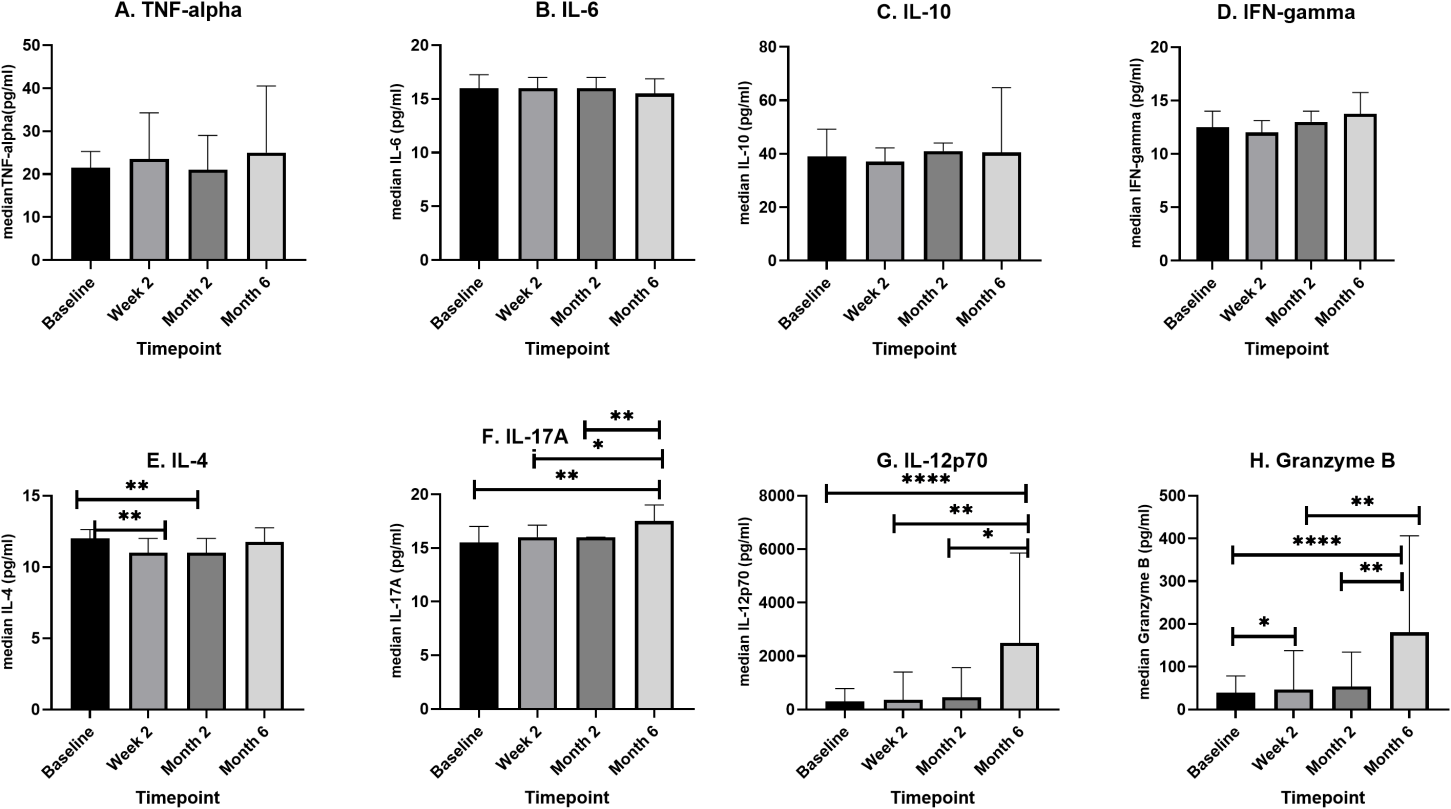
A box plot showing longitudinal changes in median cytokine levels (pg/ml) during four phases of TB treatment. Newly diagnosed TB patients (n=46) were recruited, and serum samples were taken *before initiation of TB treatment*, at the *second week of TB treatment* (n=46), at *month two of TB treatment* (n=31), and *after completion of TB treatment* (n=16). In an 8-plex Luminex assay, cytokine levels were determined for each patient at each of the four points. For each cytokine **(A–H)** median levels at each of the four time points were compared using, Kruskal–Wallis or One-way ANOVA. The box plots show the 25th, 50th, and 75th percentiles, and the whiskers represent the minimum and maximum levels of cytokine (pg/ml). Data denoting median (+IQR) values *P < 0.05, **P < 0.01 and ****P < 0.0001.

The representative bar graphs showing error bars for comparing cytokine levels at the different treatment time points are shown in **Figure 3**.

### 3.4 Diagnostic value of multiple cytokines/chemokines as differential biomarkers for active and latent TB

**Figure 4:**
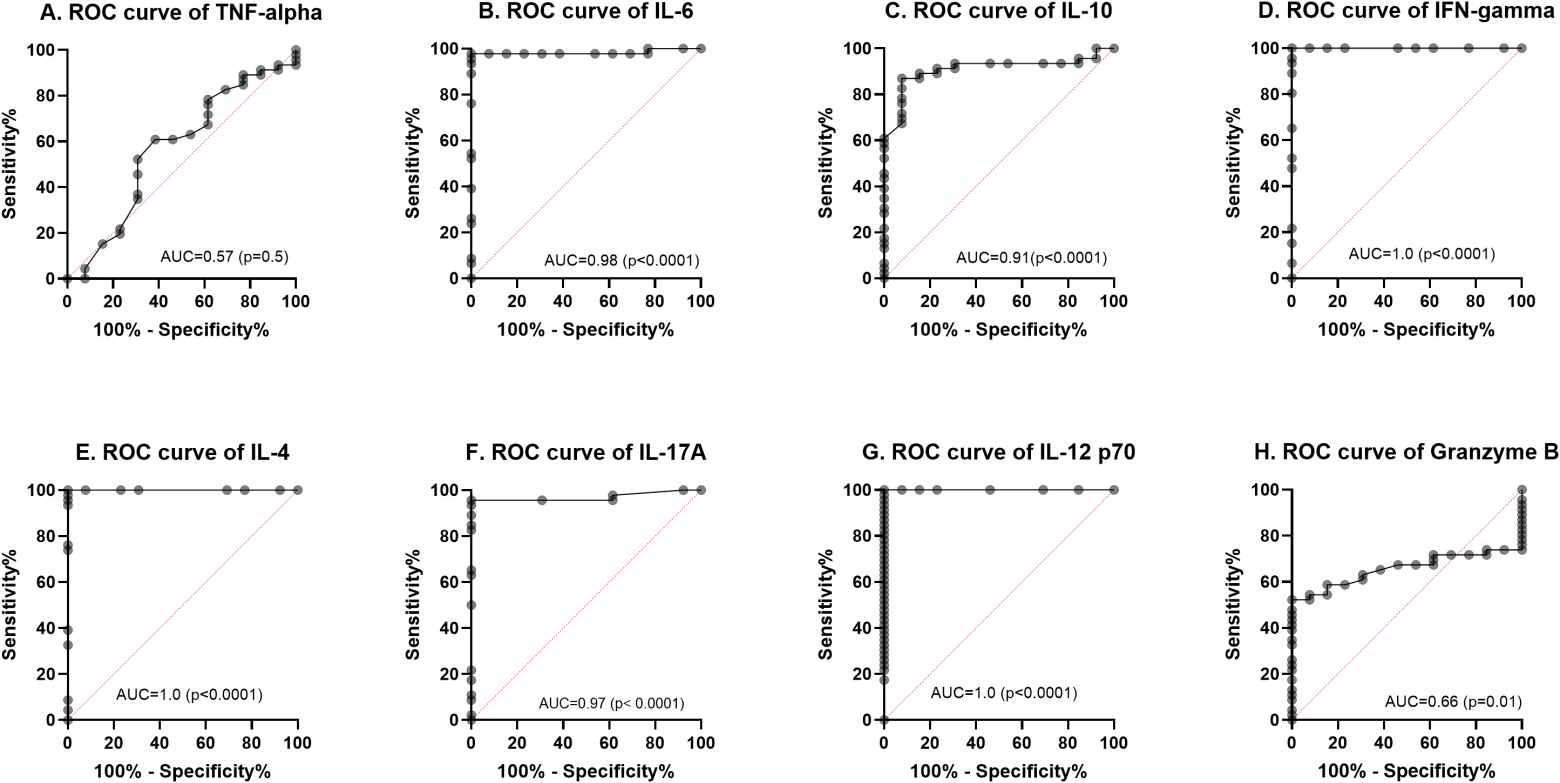
Receiver operating characteristic (ROC) curves **(A–H)** for the baseline cytokine values comparing LTBI with active TB (ATB). The solid line shows the result of absolute values of each biomarker. The area under the curve (AUC) is indicated.

A ROC curve analysis was performed to determine the diagnostic value of the eight cytokines/chemokines for ATB and LTB using % sensitivity and % specificity. The diagnostic potential was calculated from the Area Under ROC (AUROC) Curve on a scale of 0-1 (expressed as a percentage) using Graph Pad Prism (v 8.4.3) as shown in **Figure 4**. The AUROC values indicates a strong diagnostic value for IL-6, IL-10, IFN- γ, IL-4, IL-17A and IL-12p70 at 98%, 91%, 100%, 100%, 97%, 100% respectively. The ROC curve for TNF-α and Granzyme B showed a moderate predictive value with an AUROC of 56% (p< 0.4589, 95% CI: 0.3793 – 0.7562) and 66% p<0.0873 95% CI: 0.5250 – 0.7877).

## 4 Discussion

Due to the asymptomatic nature of latent tuberculosis, production of sputum for diagnosis is virtually impractical. The introduction of blood-based immunoassays, Interferon Gamma Release Assays (IGRAs) to detect latent TB infections have been useful. However, IGRAs with their detection of a single cytokine, fall short of discriminating between ATB and LTBI. We conducted this study to identify other blood-based analytes that may have this ability by comparing the levels among a cohort of ATB and LTBI cases. Among the ATB cases, there were twice as many males (69%) as females (30%) with infectivity spanning a wide age range (**Table 1**). This finding supports the view that, globally men are significantly more at risk of contracting tuberculosis than women (24). In 2022, about 5.8 million adult men were infected with tuberculosis compared to an estimated 3.5 million adult women (24). TB and its products induce the production of pro-inflammatory cytokines TNF-α and IFN-γ after stimulation of macrophages (25), and dendritic cells (26). However, TNF-α and IFN-γ are known for their auto-induction of MTB-infected macrophages and dendritic cells which, may initiate a cascade of events including chemotaxis, initiation of adaptive immunity leading to antigen-specific T cell response and subsequent granuloma formation and containment or killing of MTB. Findings of this study (**Figure 2**) suggest that TNF-α and IFN-γ responses might be useful tools for predicting active TB and as the current IGRAs are based on IFN-γ response, adding TNF-α may improve the ability for discriminating LTBI and ATB during close household contact investigation (27). Several antagonistic inflammatory cytokines: IL-6, IL-10, IL-4, IL-17A levels were higher in LTBI compared to ATB patients (**Figure 2**). The simultaneous presence suggests a coordinated response to TB infection and a possible heightened immune response during the period of dormancy than in active tuberculosis (28). IL-6 has both pro- and anti-inflammatory properties (29) and it is produced early during infection with mycobacteria which, can be found mainly at an infection site (30, 31). Thus, decreased IL-6 expression may lead to an increased susceptibility during experimental *M. tuberculosis* infection which shows that IL-6 influences the protective immune response of the host (32). IL-10 is well characterized and known for its immunoregulatory effect as an anti-inflammatory cytokine that inhibits the actions of pro-inflammatory cytokines (33). The IFN-γ levels were expected to correlate with the high levels of IL-12p70 because it is a key activator of interferon-γ producing type 1 helper (Th1) T cells (34). IL-12p70 plays a pivotal role in regulating the Th1/Th2 balance in the initial stage of immune responses. This may explain the higher levels of IL-12p70 in ATB at baseline compared to LTBI and healthy controls.

Active TB cohorts were followed at four different phases: baseline (before ATB treatment), two weeks after initiation of TB treatment, two months after initiation of TB treatment phase (intensive phase) and six months after initiation of TB treatment (completion phase). We encountered significant losses to follow up leading to only 16 of 46 ATB participants retained at the end point of treatment completion. While this may affect the generalizability of the analysis, the results provide some insights into cytokine dynamics over the course of TB treatment. From the longitudinal analysis of the levels of eight analytes in the serum during ATB, significant changes were observed for only IL-12p70 and Granzyme B (**Figure 3** **).** IL-12 is produced by activated macrophages and dendritic cells after MTB infection (35). However, increased levels of IL-12, IL-15 and IL-18 results in the stimulation of IFN-γ producing cells to produce sufficient IFN-γ (36). This initiates the auto-induction of MTB-infected macrophages and dendritic cells to activate a cascade of events including chemotaxis, initiation of adaptive immunity leading to antigen-specific T cell response (37). These series of events result in granuloma formation and containment or killing of MTB. The presence of adequate amounts of IL-12p70 in the lymph nodes ensures a sufficient production of Th1 and production of INF-γ. Effective treatment inhibits the growth of MTB in the lung thereby reducing the stimulation of inflammatory response of host to TB infection (38). More of the IL-12p70 produced is thus released into peripheral blood as the MTB is cleared in the lungs. This supports the significant increase in IL-12p70 in the plasma of the ATB cohort from baseline up to the 6 months of treatment completion observed in this study (**Figure 3**). However, Granzyme B levels increased steadily from baseline to treatment completion suggesting a possible role in host immune response to tuberculosis (39). A previous study among Ghanaian TB patients reported a significant increase in Granzyme B levels during the first two weeks in response to latency related antigen RV1733 (40). However, both Granzyme B and TNF-α showed an average predictive potential below 70% (**Figure 4**) while IL-6, IL-10, IFN- γ, IL-4, IL-17A and IL-12p70 had a significant diagnostic potential to differentiate between ATB and LTB (AUROC. 98.3%, 100%, 96.9%, 100% respectively). IL-12p70 had the highest diagnostic value (AUC 100%, p=0.0001) for differentiating between ATB and LTB. However, it may not be ideal as a biomarker for treatment response due to continuous increase over the course of treatment. It has been reported (41), that production of IL-12p70 is one of the earliest events in the activation of cell mediated immunity hence may not be an excellent predictor in advanced stages of *M. tuberculosis* infection such as in the case of multidrug resistant tuberculosis (41). IL-12p70 is also known to play a crucial role in immune reconstitution, mainly due to its ability to stimulate the differentiation of naive T cells into Th1 cells, which are vital for mounting effective immune responses against pathogens (42). The restoration of IL-12p70 and IFN-γ production is important for the recovery of the host’s ability to mount a strong immune response against pathogens and for preventing opportunistic infections. However, elevated IL-12p70 levels after treatment as observed in this study may suggest that the infection is not fully resolved, even though the patient is clinically improving. This could be due to factors like drug resistance, incomplete treatment, or the presence of latent TB infection. Further investigations on IL-12p70 dynamics following completion of TB treatment may provide more information.

Our study is limited by the small sample size of 71 participants comprising 46 active TB cases, 13 latent TB cases and 12 healthy controls hence we acknowledge that even though we have provided an insight into the performance of these 8 selected cytokine/chemokines as putative biomarkers for discriminating between active and latent TB, there is the need to confirm these findings with a larger and more diverse cohort. We used a longitudinal approach so we could assess the utility of these eight analytes as biomarkers for monitoring response to TB treatment, however due to loss to follow up, as well as other factors such as age, nutritional status, comorbidities, our data may not fully reflect individual variability in immune response.

In summary our study shows that active tuberculosis is associated with significantly higher serum levels of IL-12p70 compared to latently infected individuals and those not infected with TB. IL-12p70 also has a significant predictive potential to differentiate ATB from LTB. However, a cocktail of pro and anti-inflammatory cytokines is produced in response to tuberculosis infections. Therefore, the utility of serum cytokines in development of immunodiagnostic assays will depend on validated cut off values established from specific immune response to purified protein derivatives of *Mycobacterium tuberculosis* using a larger study cohort. We used univariate non-parametric tests due to small sample size and non-normal distributions which did not allow us to explore synergistic effects of cytokine combinations. We recommended that future studies consider using multivariate modeling to explore synergistic effects of cytokine combinations. Using a combinatorial biomarker approach (e.g., logistic regression or machine learning classifier combining multiple cytokines) could be more diagnostically powerful. Additionally, future studies could employ more recent and novel techniques such as the spatial CITE (co-indexing of transcriptomes and epitopes) sequencing, spatially resolved *in vivo* CRISPR screen sequencing via perturb-DBiT (43, 44).

## Data Availability

The original contributions presented in the study are included in the article/supplementary material. Further inquiries can be directed to the corresponding author.
