# Supplementary material for: Performance of eight serum cytokine/chemokine biomarkers in discriminating between active and latent tuberculosis infection in Ghana
Source: Front Immunol. 2025 Jun 6;16:1600712. doi: 10.3389/fimmu.2025.1600712 (PMC12179143; doi:10.3389/fimmu.2025.1600712)
Supplement: Supplementary file 1 [file Table1.docx]

Supplementary table

ROC curve analysis for ATB (n=46) and LTBI (n=13)

| **Analyte** | **Median concentration (pg/ml)** | | **Area under ROC curve** | | | |
| --- | --- | --- | --- | --- | --- | --- |
|  | ATB (n=46) | LTBI (n=13) | Area | Std. Error | 95% confidence interval | P value |
| TNF-alpha | 21.50 | 23 | 0.5677 | 0.09615 | 0.3793 to 0.7562 | 0.4589 |
| IL-6 | 16 | 31 | 0.9833 | 0.01692 | 0.9501 to 1.000 | <0.0001 |
| IL-10 | 39 | 71 | 0.9089 | 0.04055 | 0.8294 to 0.9883 | <0.0001 |
| IFN-gamma | 12.5 | 37.5 | 1.000 | 0.000 | 1.000 to 1.000 | <0.0001 |
| IL-4 | 12 | 32 | 1.000 | 0.000 | 1.000 to 1.000 | <0.0001 |
| 1L-17A | 15.5 | 23 | 0.9699 | 0.02192 | 0.9269 to 1.000 | <0.0001 |
| 1L-12p70 | 295.5 | 14 | 1.000 | 0.000 | 1.000 to 1.000 | <0.0001 |
| Granzyme B | 39 | 62 | 0.6564 | 0.06700 | 0.5250 to 0.7877 | 0.0873 |
